# Supplementary material for: Chromothripsis during telomere crisis is independent of NHEJ, and consistent with a replicative origin
Source: Genome Res. 2019 May;29(5):737–49. doi: 10.1101/gr.240705.118 (PMC6499312; doi:10.1101/gr.240705.118)
Supplement: Supplemental Material [file supp_gr.240705.118_Supplemental_file_1.zip › contigs/annotated_contigs/DB83/contig.2.DB83_length_579_mean_cov_10.4248704663.docx]

**DB83_length_579_mean_cov_10.4248704663**

```
TTCCAGCAAACATAAACCAGGCAGAAAAACGTGAAAAGGAGAGACAGGCCTAGCCTTCCAGCCTATGTCTTTCTCCCATGCTGGATGCT
 >chr9:105409139-105409398 + E=3e-144
TCCTGCCCTGGAATATCAGACTCCAAGTTCTTAGACTTCTAGACTTGGACTGGCTCTCCTTGCTCCTCAGCTTGCAGGCAGCCTATTAT

AGGACCTATTGATCATGTAAGTTAATACTTAATAAATTCCCATATATATATATATATATATATATATATATATATATAT|AT|GTATCT
                                                                                >chr9:1
GAGAAAATTTAATATAGACTGGAACTAGATAACAAGAAGTATATTTATATTTTAGATATAAAAATGACTGGTTATATACAAAATAGTAT
05409931-105410253 + E=4e-182
TCTTATACTGCAGATAACTTACTTACAAAATTCATTTTAATTAAAACCAGGAATTAGAATAATAGTAAAATATTTCATGCTAGAATTTT

AGATTATGTTGGTCAGTTTTATTTATTCTCATTTTTGTTTTGTTTCTTATTTTTACATATGCCATTAAATGGAGGAAAGTATGACACAA

TGCTAGTAGTATCTTCAGGTAATTGTTTCAAAATTGGTTCTAGTGAA
```
